# Health Effects of Whole Grains: A Bibliometric Analysis

**DOI:** 10.3390/foods11244094

**Published:** 2022-12-18

**Authors:** Xun Wei, Wei Yang, Jianhui Wang, Yong Zhang, Yaxuan Wang, Yan Long, Bin Tan, Xiangyuan Wan

**Affiliations:** 1 Zhongzhi International Institute of Agricultural Biosciences, Shunde Innovation School, Research Center of Biology and Agriculture, University of Science and Technology Beijing, Beijing 100024, China; 2 Beijing Beike Institute of Precision Medicine and Health Technology, Beijing 100192, China; 3 College of Basic Science, Tianjin Agricultural University, Tianjin 300384, China; 4 Academy of National Food and Strategic Reserves Administration, Beijing 100037, China

**Keywords:** whole grains, nutrition, disease, healthy diet, bibliometrics

## Abstract

Whole grains have been recommended in the diet in most countries, with numerous publications focusing on their health effect. A systematic analysis of these publications on different research methods, regions and perspectives will contribute to an understanding of the innovation pattern in this field. This bibliometric study analyzes the global publication characteristics, hotspots and frontiers of whole grain health benefit research, and discusses the trends and prospects of this topic. The overall number of publications is on the rise, with the United States contributing the most publications. The most cited literature shows that observational studies, systematic reviews and meta-analysis are the most widely used methods. The main focus in this area is on dietary fiber and bioactive substances, while the latter has received increased attention in recent years in particular. With the increasingly prominent problems of hidden hunger and chronic disease, the development of whole grain foods and their optimum intake have gradually become hot topics. In addition to the need to reveal the mechanism of whole grain health effects, consensus needs to be reached on standards and definitions for whole grain foods, and attention should be paid to the retention of taste and healthy nutrients in processing.

## 1. Introduction

Chronic metabolic diseases have been recognized as worldwide problems of human health, and the non-negligible causes of the global burden of disease (GBD) as well [1]. Medical services are required to keep improving, resulting in increased public health costs, which can be alleviated by adding whole grains to the diet. Compared with refined grains, whole grains retain more beneficial ingredients including dietary fiber, vitamins, minerals and phytochemicals, which show effective functions for health [2]. Evidence from epidemiologic data has suggested that whole grain intake is associated with reducing risk of obesity [3], cardiovascular disease (CVD) [4], type II diabetes [5] cancer [6] and other chronic diseases [7,8], based on the function of diet fiber, resistance starch (RS) and other bioactive components. Grains are promoted in 90 countries around the world with clear dietary guidelines, with 44% of countries specifically encouraging whole grains [9].

Whole grains are composed of intact, milled, cracked, or flaked grains, with endosperm, germ, and bran presenting in the same proportion as primary grains, and this basic definition of whole grains issued by the American Association of Cereal Chemists (AACCI) is recognized worldwide [10,11]. It is precisely the difference in processing that makes whole grains retain more nutrients and active substances from bran and germs than refined grains [12]. The potential physiological mechanisms of the health function of whole grains are not fully understood, but are most likely due to the synergistic effects of dietary fiber which is mainly concentrated in the bran portion, and various phytochemicals [13]. Recent studies have found that dietary fiber can not only shape the human intestinal flora [14] and be fermented by Firmicutes in the intestine, but also interact with the intestinal mucosa to promote homeostasis [15]. Other components showing great potential for nutrition and activity such as antioxidation are phenolic compounds, benzoic acid, cinnamic acid derivative, lignan and other phytochemicals [16]. They are degraded, metabolized and transformed into a series of important metabolites such as SCFAs by intestinal microflora, and further affect cellular metabolism [13].

Whole grains have great potential for the treatment and prevention of epidemic diseases. The research progress and trend of whole grain health effects can be analyzed more comprehensively through bibliometric tools. To date, bibliometric analysis has received scant attention in the research of whole grain effects. Therefore, this paper used the method of bibliometrics to handle relevant literature data from 2000 to 2021, and analyzed the development process and trend of research on the health effects of whole grains. Differently to previous review articles which explain important concepts and advances in a certain field, the present study focused on a broad analysis of whole grain health effects to identify the hotspot. Finally, a deep discussion on the prospects and challenges have been provided to support future research and decision-making in the field of whole grain promotion.

## 2. Methods and Data

The systematic development of bibliometrics is largely attributed to its founders D.J.D. Price and Eugene Garfield, who have an important influence in the field of library and information science [17]. In recent years, bibliometrics has gradually been accepted as a useful tool in different professional fields, where the research trends and gaps can be analyzed, and is helpful to comprehend the prospects and characteristics of the topic [18,19].

Web of Science is a quotation index database of global authority, currently including more than 8800 international high-level journals. Numerous bibliometric analyses are based on this database, which has a large amount of literature on this topic. The bibliometric study used scientific output data from the core collection of Science Citation Index Expanded (SCI-E) in Web of Science and was performed as presented in Supplementary Figure S1. We searched for literature on October 1, 2022, through a search strategy based on keywords related to health effects of whole grains (see Supplementary Table S1). This study only included reviews and articles from 2000 to 2021, and non-English literature was not considered. Duplicate publications were also excluded.

The data from the documents obtained since the 21st century were exported and analyzed on bibliometric software. Citespace (5.6.R1) was used to visualize network maps of countries and institutions, as well as the timeline map of keywords and keyword bursts. The “Bibliometrix” package (R language) was used to obtain a keyword cloud map, three-field plot and thematic map that was adjusted via an online platform (https://sankeymatic.com/build/, accessed on 13 December 2022). Keyword clustering information generated by VOSviewer (1.6.18). Considering disciplinary developments in the context of bibliometrics, the constructive views and findings were given. In addition, in order to obtain comprehensive and recently updated information, some references were consulted for more detailed analysis.

## 3. Research Tendencies and Progress Analysis by Bibliometrics

The results of the bibliometric analysis on health effects of whole grains are presented as follows, indicating country/region, institutions, journals, the most prominent research areas and author keywords. Each part of the results was discussed to provide insightful data on research progress and trends on the topic.

### 3.1. Evolution of Publications and Regional Distribution

**Figure 1:**
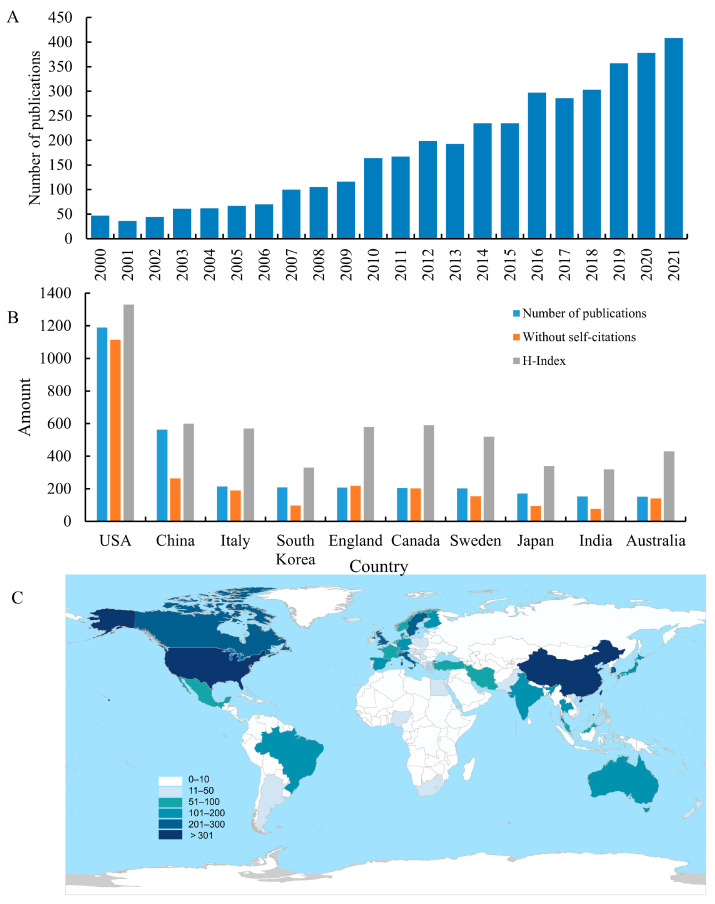
Evolution of publications and contributions of different countries/regions. (**A**) Annual changes in the number of publications. (**B**) Number of publications, without self-citations (actual value/60), H-index (actual value ×10). (**C**) Regional clustering characteristics of publications.

From 2000 to 2021, the amount of literature on the health effects of whole grains showed an overall upward trend, although the amount of literature may decline slightly in some years or remain unchanged in the previous year, as shown in Figure 1A. Figure 1B,C shows that the United States leads the world in the number of publications with high citations and H-index, reflecting the high quality of research. Similarly, Italy, England, Canada, Sweden have higher H indexes, indicating a higher level of research, which may be due to the increased interest in the health effects of whole grains and the emphasis on healthy diet in rich countries. Although China ranks second in the number of publications, the quality of related research needs to be improved.

### 3.2. Collaboration and Competition in Research

**Figure 2:**
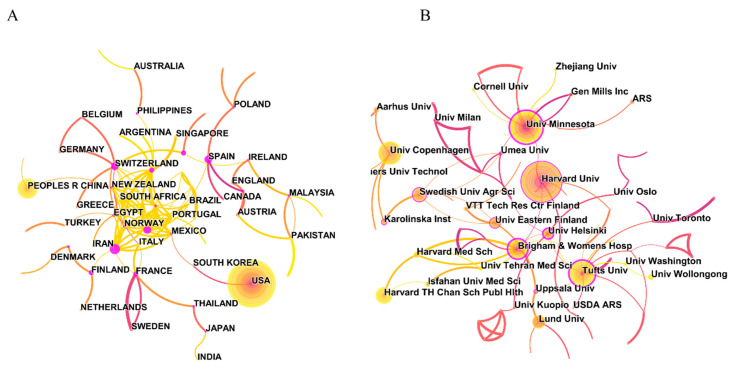
Collaborations of (**A**) countries/regions and (**B**) institutions for studies on the health effects of whole grains.

The United States and China, which have a high number of publications, have a low degree of centrality in the cooperation network (Supplementary Table S2). As shown in Figure 2A, the annual ring-shaped country node represents the year of publication with a gradient color, and the annual number of documents is expressed in different widths. Therefore, countries with larger nodes have more total publications. The state node with an external purple ring has greater centrality and extensive cooperation with other members. The colors of the links between countries, from purple to yellow, indicate the evolution of cooperation time, and the width represents the closeness of the relationship. In contrast to the United States and China, Denmark, Norway, France, Australia, and England have extensive cooperation with other countries/regions, although the number of publications is not significant. In particular, Denmark and Norway have conducted extensive exchanges with other countries in the study of whole grain health effects in recent years. Indeed, Scandinavian countries have a long tradition of eating whole grain foods, crisp, black bread made from whole grain rye and wheat, and assorted cereals containing whole grain oats [20].

The ranking of most productive institutions in terms of an absolute number of publications brings Harvard University into the first position, followed by the University of Minnesota and Tufts University (Supplementary Table S3). Harvard University and Tufts University have carried out extensive cooperation with other institutions in research, while the University of Copenhagen and Brigham and Women’s Hospital are also significant contributors, with the latter having more collaborators (Figure 2B).

### 3.3. The Distribution of Research Hotspots

The distribution of journals that notice the health effects of whole grains was relatively differential, with only 5.0% of all papers published in *Food Chemistry*, which tops the list with 195 citations and 11,550 citations. Although *The American Journal of Clinical Nutrition* ranks fourth in the number of publications, it shows good strength in citation times, H index and impact factors (Table 1). One of the most important indicators to measure the hot topics in a professional field is the most frequently cited literature. Table 2 shows information of the most cited literature from 2012 to 2021. Studies on the health effects of whole grains have mainly used the methods of systematic reviews and meta-analyses. Because of the pressure for timely, informed decision-making in public health and clinical practice and the explosion of information in the scientific literature, research findings must be integrated. Systematic review and meta-analysis are crucial methods in evaluating the effectiveness and safety of medical interventions and observational studies of the effects of healthy diets [21]. This widely accepted statistical analysis approach requires rigorous steps to make its conclusions more accurate [22].

The most frequently cited literature, published in 2019 from *The Lancet*, is a systematic review which assessed the consumption of major foods and nutrients in 195 countries and found that the low intake of whole grains have a significant impact on morbidity and mortality of non-communicable diseases [23]. Similar to the most cited literature concerns, the tenth-ranked study also focused on the relationship between diet and disease risk [24], which finds that high intakes of whole grains, fruit, vegetables, also have important preventive and health benefits. A prospective study conducted by Ananthakrishnan et al., ranked ninth, found that fruit fiber was associated with a lower risk of Crohn’s disease, but whole grains or other soy fiber had no significant improvement in the risk of bowel disease [25]. Other highly cited articles focused on the benefits of dietary fiber to the gut [26,27], the effect of whole grains on the risk of cardiovascular disease [28,29,30], coronary heart disease [28], type 2 diabetes [29,30,31], cancer [24,28,29,32] and the capacity to adjust level of body weight [29,30], blood pressure [29], cholesterol [29]. In general, the research objects of the top 10 cited publications do not involve animals, and observational studies, systematic reviews or meta-analyses based on humans play an important role in this topic. However, there is little literature on the health mechanism of whole grains, with only one article and one review reporting that the intestinal environment was improved.

**Figure 3:**
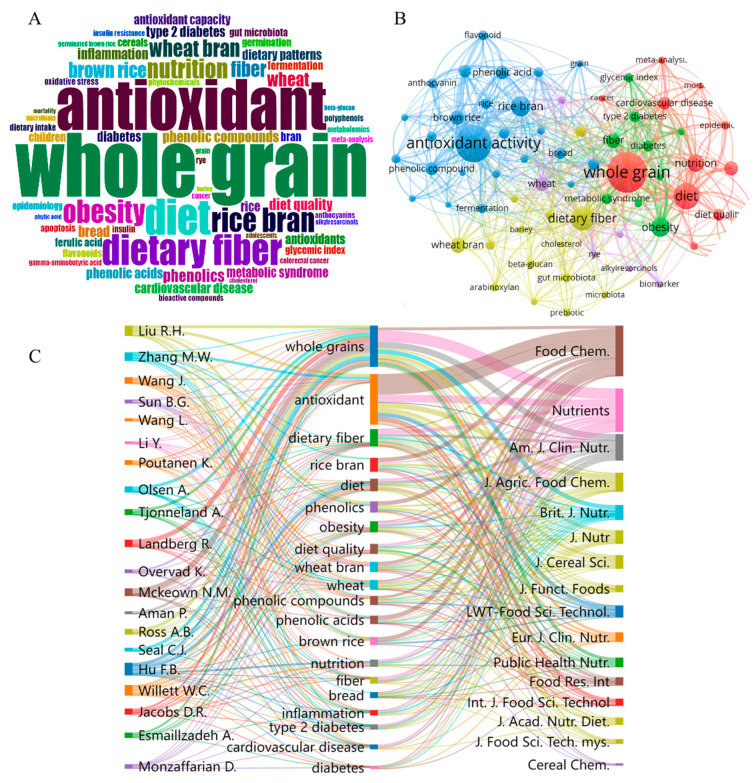
Distribution of hotspot in research on health effects of whole grains. (**A**) Co-occurrence of keywords (**B**) Cluster of keywords, the colors represent different clusters, a total of 5 categories were found. (**C**) The three-fields plot correlating the top 20 authors, author keywords, and sources.

As shown in Figure 3A, the main keywords of whole grain health research are whole grains, antioxidants, dietary fiber, bran, obesity, nutrition, cardiovascular disease, type 2 diabetes, phenolic acids and phenolic compounds, etc. VOSviewer software was used to analyze the literature data, and the generated clustering diagram is shown in Figure 3B. Research on the health effects of whole grains has focused on the following categories: antioxidant studies (blue), dietary intake and nutrient composition of whole grains (red), benefits of dietary fiber (yellow), therapeutic effects on diseases such as obesity and diabetes (green), phytochemicals and biomarkers of whole grain intake and altered biochemical markers (purple).

Figure 3C shows the interconnections among authors, author keywords, and sources. The primary direction of whole grain research is dietary nutrition, and antioxidant is an important index to evaluate the good quality of whole grain. Studies have focused on major components including dietary fiber, rice bran, phenolic compounds and major types including wheat bran, wheat, brown rice, and whole wheat bread, as well as their effects on inflammation, diabetes, and cardiovascular disease (CVD). Research of Dr Hu and Dr Willett focused on the influence of diet/lifestyle, metabolic and genetic factors on epidemics such as obesity, type 2 diabetes and CVD. Swedish scientist Rikard Landberg studied the effects of foods and food components on health and disease risk through observational, intervention studies and various model systems. *Food Chemistry* is listed as the most important journal here, covering different directions in terms of whole grains, antioxidants, nutrition, disease, etc., while other journals are unsurprisingly devoted entirely to food nutrition, science and health.

### 3.4. Evolution of Research Trends

**Figure 4:**
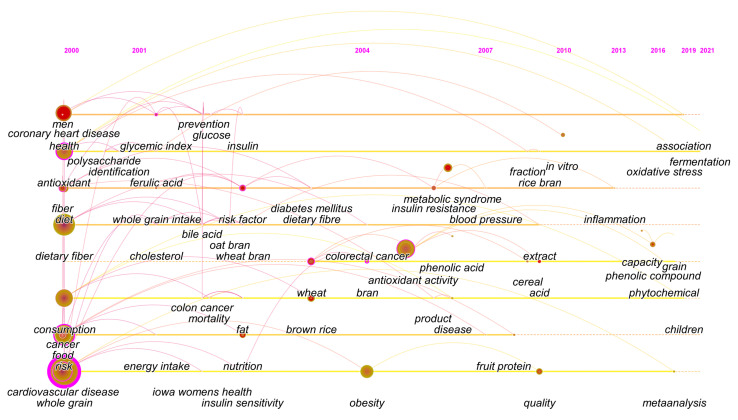
The timeline of keywords evolution.

The evolution of the keyword timeline is shown in Figure 4. Over the past few decades it has long been concerned with the nutritional benefits of whole grains, which are rich in dietary fiber and bioactive substances, to improve the dietary quality that has become a scientific concern in recent years, as also shown in Figure S2A. For decades, people have generally paid attention to the effects of whole grains on obesity, diabetes, cardiovascular disease, cancer, metabolic syndrome and other diseases, among which the keywords burst map (Figure S2B) shows that coronary heart disease appears earliest and has the most frequent duration (34.75). Studies have shown that whole grains can effectively balance a child’s diet [33,34] in which school meals may play an important role [35]. Similar to past observational studies, more evaluations of the health effects of whole grains, such as meta-analysis, have emerged (Figure 4, Figure S2A). Current studies evaluating the health effects have used statistical models to assess the association between whole grain consumption and risk of disease, and the results are generally consistent with existing public health recommendations. Studies on phytochemicals of whole grains have already begun [36,37], and the evaluation of antioxidant capacity of phenolic compounds has gained further attention in recent years. It is important to note that the proper treatment of grains can keep the active substances basically intact and facilitate the development of highly active whole grains, which can have a more significant effect on their health effects.

The evaluation of the content and activity of various components according to different types of whole grains is conducive to a clear and systematic understanding of the health effects of whole grains, and contributes to finding the biological mechanism of whole grains to reduce the risk of several diseases, such as the regulation on inflammation as a mediator of disease [38]. While the anti-inflammatory effects of whole grains have not been conclusively established, there are reasons to believe in the potential of whole grains to combat inflammation [39]. In addition, Figure S2A adds that fermentation and gut microbiota are the other topics that have been studied more in recent years and cannot be ignored.

**Figure 5:**
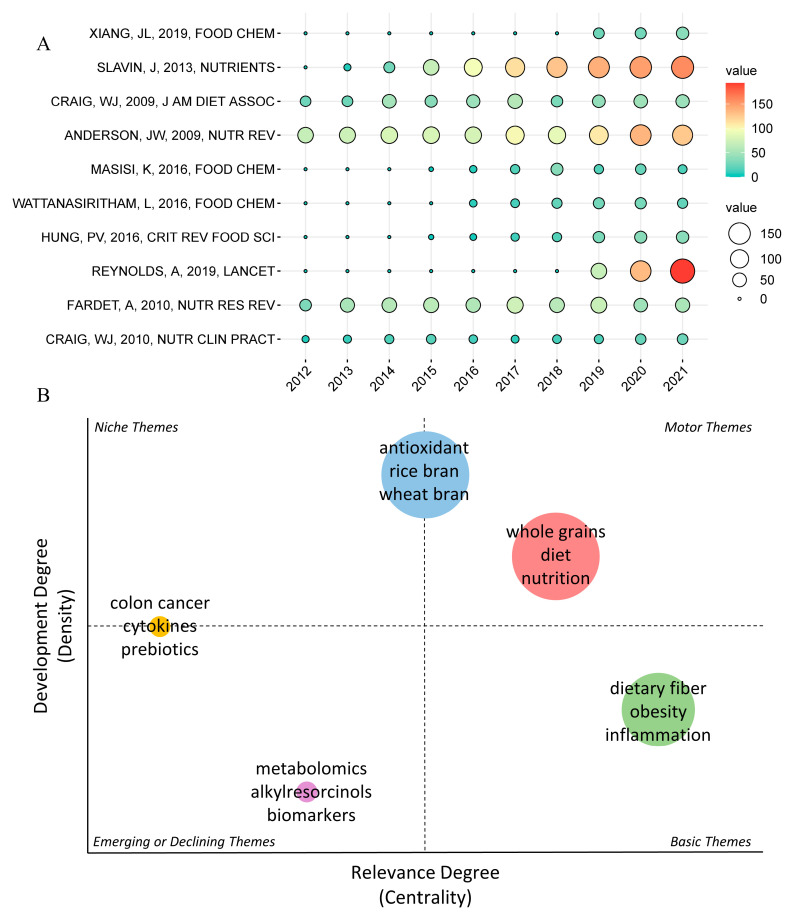
Evolution of research trends. (**A**) Top 10 publications by usage count over the last ten years. The size and colors of the circle represent the citations of papers. (**B**) Thematic map.

Usage count directly reflects the interest and intention of the majority of scientific researchers when obtaining literature, the selection of professionals in the field of whole grain effects can be seen from it, and the current or potential hot areas can be distinguished by combining with the citation frequency of a paper (Figure 5A). Slavin [26] and Reynolds [29], who focus on the health benefits of dietary fiber, also rank second and fourth, respectively, in the most cited papers (Table 2), except for a review from Anderson [40] with a high usage count. Based on these three papers with high usage count and citations in recent years, it can be found that professionals have a high interest in dietary fiber and that whole grain products rich in dietary fiber are beneficial to the treatment and prevention of chronic or metabolic diseases. Studies have shown that dietary fiber is a prebiotic to specific microorganisms and is important for gastrointestinal and human health by regulating the intestinal flora [14,41], which can significantly reduce the risk of coronary heart disease, stroke, hypertension, diabetes, obesity and some gastrointestinal diseases [40]. Slavin [26] summarizes the effects of different classifications of dietary fiber on disease risk factors and intestinal probiotics, although insufficient dietary fiber intake may weaken this health benefit. Resistant starch may alleviate the problem of inadequate dietary fiber intake to some extent. Because its color, taste, texture are similar to starch, as an additive or directly as a food, it will not affect the taste and flavor of the food itself, which effectively solves the defects of ordinary dietary fiber [42]. At the same time, it also has a functional component, which has become a research hotspot in the food processing industry. Other articles in the top ten of the usage count, especially those that appeared in the previous five years, do not have prominent citations, despite the high number of views. It is worth noting that a study on the phenolic compounds and antioxidant activity of finger millet ranked first in terms of the usage published in 2019 [43], which may be a non-negligible topic even though citations are not brilliant.

According to the relevance and development degree of the topic, the thematic map is divided into four quadrants, (i) motor themes, (ii) basic themes, (iii) emerging or dominating themes and (iv) niche themes. Studies on dietary fiber, obesity, and inflammation are likely to be further developed due to their low density and high centrality. Metabolomics in the study of the biologic mechanisms of whole grains and biomarkers of intake, which are both in the third quadrant, may be further developed in the future. Research in the field of antioxidants seems to be distinct from research on the health effects of whole grains, with the former being more oriented towards chemistry and biology, and thus having a high density but medium centrality, while the latter is most often concerned with topics in the medical direction of diet, nutrition and epidemic diseases (Figure 5B).

## 4. Progress and Future Direction of Whole Grain Health Effects

### 4.1. Research Gaps Need to Be Filled on Molecular Level and within Different Subtypes of Whole Grains

The health benefits of whole grains are generally widely recognized, especially since there are a large number of observational studies based on humans that show a significant benefit of whole grains on epidemic diseases, although they have not been thoroughly studied on the molecular level. The inescapable intermediate factor in the health benefits of whole grains is the gut. The effect of processing was greater than that of grain type, with extruded whole wheat and brown rice worsening the metabolic health of the host compared to whole wheat porridge [44]. This is due to their different impacts on the gut microbiome, so more comprehensive analyses of the health effects of more different types of whole grains processed in more different ways are needed to further guide the food industry to consider more urgently the development of processing strategies that address the microbiome and the public health crisis caused by poor diets. In addition, probiotics have been shown to be effective in regulating blood glucose homeostasis [45], improving the symptoms of colon cancer and intestinal damage [46], and promotes the longevity of *C. elegans* [47]. For target probiotics, there is great potential to develop foods with beneficial components of whole grains as prebiotics. However, another study showed that a whole grain diet did not alter insulin sensitivity or the gut microbiome, even though whole grains significantly reduced energy intake and body weight and circulating markers of inflammation in adults at risk for metabolic syndrome [48]. Differences between studies may be due to differences in study individuals, intervention controls, and methods, and therefore require more detailed evaluation of different subtypes of whole grains, although overall higher intake of whole grains compared to refined grains is associated with improvement and maintenance of health on different indicators. Reynolds et al. found [29] that eating more whole grains and foods rich in dietary fiber may help prevent obesity, heart disease, diabetes, cancer and reduce the risk of premature death. However, the carbohydrate of whole grains is not lower than that of refined grains, and either too much or too little dietary carbohydrate can increase mortality [49]. Therefore, replacing refined grains with whole grains may have health benefits, while overconsumption of carbohydrates may lead to adverse consequences. Incorporating the remaining whole grains with the endosperm removed into the diet seems to be a viable way to control carbohydrate intake. 

As an important ingredient in whole grain foods, sprout whole grains are receiving increasing attention. Germination technology can further improve the nutritional value and bio-efficient utilization of whole grain products [50,51]. At present, there is no unified standard for grain germination, and the processing standard for germinated whole grains and the evaluation of the active ingredients and their health effects after germination need to be improved. In addition, more research is needed on the safety of whole grains, as wheat contains many other ingredients besides gluten that can cause symptoms, including inhibitors of α-amylase and trypsin (ATIs), lectins, and rapidly fermentable carbohydrates (FODMAPs) [52]. Detailed characterization and quantitative analysis of grain components based on the availability of high-quality standard materials are needed to support the elimination of undesirable components through food processing or targeted breeding including gene editing. The molecular analysis and editing of the cereal development regulation network is not only helpful for the production of drugs using plants as bioreactors, but also improves the yield of beneficial ingredients and the development of functional foods [53,54]. In addition, pollutants such as mycotoxins and pesticide residues, which are mainly present in the bran and germ of grains, should also be considered. Therefore, the application of pesticides in line with health standards is also required to produce healthy and safe whole grain foods.

### 4.2. Scientific Consensus on Whole Grains and Health Should Be Boosted Based on Epidemiological and Intervention Studies

Hidden hunger refers to the presence of multiple micronutrient deficiencies (particularly iron, zinc, iodine and vitamin A), which can occur without insufficient energy intake due to energy-intensive but malnourished diets. It is estimated to affect more than two billion people worldwide, especially in low- and middle-income countries that rely on low-cost staple foods and have limited dietary diversity [55]. Contrary to research in the field of food science [56], Godecke et al. found that the supply of cereals is less helpful than meat, eggs, milk, rhizomes, fruits and vegetables in reducing the burden of chronic and hidden hunger [57]. The possible reason is that cereals are primarily a source of calories for the poor, and there is little concern about the retention of their nutrients, although high levels of cereal production and imports help to alleviate chronic and invisible hunger [56]. Nutrition helps to reduce the risk of disease, but it seems that people are gradually aware of the problem of invisible hunger with unbalanced nutritional intake. This invisible hunger has an aggravating effect on the development of human chronic diseases such as obesity and chronic diseases in the elderly [58].

Cereals are an important source of dietary fiber, plant protein, phytochemicals, and essential vitamins and minerals [59]. Due to the imbalance of global economic growth and regional development, people’s diet is also developing in a diversified direction and tends to eat energy-dense foods rich in refined carbohydrates and fats and low in dietary fiber (DF). An example of this negative shift is the increasing use of whole grain shelling and finishing to obtain refined grain products, resulting in reduced intake of dietary fiber, micronutrients and phytochemicals in the human diet [13]. Fortunately, people have noticed this phenomenon of increasing the burden of invisible hunger and disease. Future policy reforms need to provide incentives and information to help encourage manufacturers to increase the supply of whole grain products, while reducing the supply of refined grain products and encouraging consumers to shift from refined grain products to whole grain products [60,61].

Obesity has become a global epidemic and public health crisis. Nearly two billion adults worldwide are considered overweight, and more than half of them are classified as obese [62]. The number of people with diabetes which is closely related to obesity is estimated to reach 578 million by 2030, and this number may grow to 700 million by 2045 [63]. Cardiovascular disease (CVD), the leading cause of death worldwide, will cause 23 million deaths per year by 2030 [64]. 

Whole grain food can affect body weight and satiety. Studies have shown that coarse grain consumption is negatively correlated with metabolic syndrome [65]. In epidemiological and intervention studies, whole grain consumption has generally been shown to be positively associated with reduced indicators of overweight and obesity [3] and to be beneficial for waist circumference in overweight children [66]. A randomized controlled trial has shown that eating coarse grains has a certain role in promoting glucose metabolism in diabetic patients [67]. Because resistant starch found in whole grains is not digested in the small intestine, it does not increase glucose, and thus has an effect on glycemic index and diabetes treatment and prevention [68]. As a prebiotic in the large intestine, resistant starch can also regulate intestinal bacteria and thus improve blood sugar [69,70,71]. Other benefits of resistant starch include increased satiety [72], treatment of constipation [73], reduction of cholesterol [74], and a lower risk of colon cancer [75] is also being further explored. The hypoglycemic effect of Foxtail millet (FM) may be partially mediated by intestinal flora, and there is a dose-dependent relationship with the improvement of blood glucose metabolism [76].Studies have shown that the increase of whole grain intake is related to the reduction of the risk of gestational diabetes mellitus (GDM) [77]. High dietary fiber intake is associated with reduced risk of cardiovascular disease (CVD), and increased dietary fiber intake has been shown to reduce blood pressure and other cardiac metabolic risk factors. Recent meta-analysis has shown the potential benefits of increasing dietary fiber intake in patients with CVD and hypertension [78]. Chen et al. found that due to low whole grain intake, men and the elderly have a higher risk of cardiovascular disease death. Increasing the intake of coarse grains may have a better preventive effect on CVD, and effective strategies are needed to increase health awareness. 

With the rapid growth and aging of the global population, the risk of cancer as the leading cause of death is increasing. In the face of these epidemic diseases, dietary prevention and adjuvant therapy is an effective and safe means. Encouragingly, whole grains have been shown to prevent chronic diseases and promote health through numerous studies [79,80]. Unlike refined similar products, whole grain intake is negatively correlated with the risk of gastrointestinal cancer and is most consistent with the risk of colorectal cancer [81]. Coarse grain is the most basic and important food source of bioactive phytochemicals. There is a negative correlation between coarse grain intake and the incidence of breast cancer, which has a clear role in all stages of breast cancer [82]. The meta-analysis of cohort and case–control studies consistently found [6] that whole grain intake was associated with a lower total cancer and site-specific cancer risk, and supported the current dietary recommendations for increasing whole grain intake.

### 4.3. Standard and Recommended Intakes Are the Key to the Health Effects of Whole Grain Foods

Whole grains are part of a healthy diet pattern, with many benefits, and are recommended in many countries. However, many countries are facing the problem of insufficient whole grain intake, and few countries have standardized the specific intake of whole grains. Even in countries with intake guidance, recommendations may be vague and qualitative. The development of uniform standards for whole grains and their foods encourages health and regulatory authorities to make sound recommendations and management for public health benefits [20]. Whole grains in percentage and grams or a combination of both have been used by different regions and health promotion organizations. A minimum of 51% or 8 g of whole grains per serving is often cited as the minimum qualifying limit [10]. According to the Dietary Guidelines for Chinese Residents (2016), it is recommended to eat 50–150 g of whole grains and bean foods every day, with the former accounting for 1/3–1/4. The Dietary Guidelines for Americans (2020–2025) recommend eating 6 ounces of grains throughout the day, with at least 1/2 whole grains. The Australian Dietary Guidelines recommend 4–6 servings (about 120–180 g) of grains throughout the day, preferably mostly whole grains. The Canadian Dietary Guidelines do not recommend specific amounts, but emphasize that whole grains should be eaten every day.

Whole grain intake and its definition need to be standardized worldwide, which is conducive to the accuracy of scientific research. Compared with people who eat the least, people who eat the most whole grains have a lower risk of chronic diseases such as cardiovascular disease and diabetes. This finding is one of the most consistent findings in nutritional epidemiology. However, in observational studies and meta-analysis studies, the exposure of whole grains is not uniform and standardized, which hinders the study of the health benefits of whole grains, may play a potential interference factor, and is not conducive to dose–response effect analysis. Korczak et al. proposed to quantify the amount of whole grains in food or products in grams based on dry weight, and the definition and processing of whole grains should be described in detail as much as possible [83]. Another important indicator is the intake of biomarkers in whole grains, which is of great value for scientific research [84].

The whole grain diet is facing many challenges. In addition to the relevant laws and the definition of whole grain food standards, changing existing eating habits is a difficult problem for the implementation of whole grain. Proposals to increase dietary fiber and whole grains have been promoted for years, but little progress has been made in changing consumer behavior. Currently, whole grain intake is more likely to be driven by traditional food consumption in a given country than by consumers who are on diet recommendations or seeking whole grain health benefits [20]. Consumers seem to have become accustomed to delicately processed foods. Although these foods lose many nutrients, their taste and flavor are popular. Most of the progress in moving consumers from refined grains to whole grains has been achieved by incorporating whole grains into already popular foods, such as breakfast cereals, breads and snacks. However, many of these foods are considered super-processed [85]. Therefore, nutritional stability and palatability need to be considered in the processing and production of whole grain foods.

### 4.4. Breaking the Negative Correlation between Taste and Nutritional Value Plays Important Role in the Promotion of Whole Grain Foods

A survey of a group of British adolescents found that the main barriers to the promotion of a whole grain diet were public difficulties in identifying whole grain products and their health benefits, poor taste and visual appeal, and poor availability outside the home [86]. Compared with refined grains, the taste and texture of whole grains determine to some extent all the public choices, even if they know the health benefits of whole grains [87]. The popularization of scientific knowledge of the health benefits of whole grains and the easier access to food are the current directions of national and public health agencies, and research on the taste of whole grain foods is also ongoing. Health agencies are also paying particular attention to the dietary health of women, infants, and children. In discussions of the Special Supplementary Nutrition Program, it is mentioned that the cost of whole grain foods and consumer choice based on taste and cultural preferences are essential for the implementation of whole grain [88]. A complementary feeding period is the key to shaping infant food preferences and habits. The introduction of whole grain cereals at the appropriate stage of life so that whole grains are accepted throughout the life cycle also requires attention to infant safety and taste acceptance. In college canteens offering blueberry muffins with different proportions of whole wheat flour, 66% of students increased their preference for muffins with 100% whole wheat flour [89]. Different from the traditional eating habits of whole grain foods in countries such as Denmark, a study based on dietary acceptability and regional characteristics in sub-Saharan Africa showed that biscuits made with whole sorghum flour and insect flour (3:1) instead of 20% of the added amount of wheat flour had improved taste and nutrition [90]. This shows that the compromise method of making food with different whole grain addition ratios is available. The taste preferences of consumers may also be affected by visual preferences. Smith et al. [91] determined the optimized bread coloring scheme through the response surface method to make whole wheat bread more uniform in appearance, and through the color optimization of whole grain food, it is beneficial for consumers to choose the whole wheat bread that looks better. Therefore, the mixing of whole grain and refined grain food is a way to solve the problem of insufficient whole grain intake. In addition, controlling germination has a significant impact on the function and flavor of wheat flour, and foods made from germinated whole grains seem to be more popular [92]. Overall, in addition to the need for national and health authorities to popularize the health benefits of whole grains, push for standard definitions, and make whole grain foods more accessible, more research is needed to improve the flavor to increase popularity.

## 5. Conclusions

This paper analyzed the progress and trend of the research on the health effects of whole grains by means of bibliometric analysis. Since 2000, there has been a significant increase in the literature on the health effects of whole grains, with increased emphasis on whole grain health research in relatively wealthy areas such as Europe, East Asia, and North America. The main methods of studying whole grain intake and health benefits are observational, interventional and through meta-analysis. However, studies at the molecular mechanism level need to be further developed and updated. In recent years, the focus has been on diet quality, inflammation, antioxidant properties, disease risk, and grain processing, and studies of bioactive substances should not be ignored. Whole grain health mechanisms and safety need more systematic and comprehensive evaluation. Considering the hidden hunger and chronic disease burden worldwide, whole grain research should also focus on the area of rulemaking and processing to contribute to healthy whole grain food promotion to meet the health criteria of intake recommendations.

## Figures and Tables

**Table 1:** The top 10 most active journals.

Rank	Journal	Np ^1^	Nc ^2^	H-Index	IF (2021)
1	*Food Chem.*	195	11,550	62	9.231
2	*Nutrients*	168	4479	28	6.706
3	*J. Agr. Food Chem.*	135	9614	56	5.895
4	*Am. J. Clin. Nutr.*	115	13,917	64	8.472
5	*J. Nutr.*	111	6502	44	4.735
6	*Brit. J. Dermatol.*	95	4851	37	4.125
7	*J. Cereal. Sci.*	87	3898	33	4.075
8	*LWT-Food Sci. Technol.*	80	2238	27	6.056
9	*Eur. J. Clin. Nutr.*	72	3418	34	4.884
10	*Cereal Chem.*	67	1022	20	2.534

^1^ Np: Number of publications; ^2^ Nc: Number of citations.

**Table 2:** Top 10 most cited articles in the whole grain health effect research.

Ranking	Title	Issue	Journal	Citations	Year
1st	Health effects of dietary risks in 195 countries, 1990–2017: a systematic analysis for the global burden of disease study 2017	This study evaluated the impact of dietary risk factors on the mortality rate of non-communicable diseases, including high sodium, low whole grain, low fruit intake.	*The Lancet*	1607	2019
2nd	Fiber and prebiotics: mechanisms and health benefits	This review summarizes the benefits of dietary fiber as prebiotics to promote the reproduction of gastrointestinal probiotics and maintain human health.	*Nutrients*	999	2013
3rd	Whole grain consumption and risk of cardiovascular disease, cancer, and all-cause and cause-specific mortality: systematic review and dose-response meta-analysis of prospective studies	This meta-analysis demonstrates that whole grain intake is associated with reduced risk of coronary heart disease, cardiovascular disease and total cancer and all-cause mortality.	*BMJ-British Medical Journal*	538	2016
4th	Carbohydrate quality and human health: a series of systematic reviews and meta-analyses	Prospective studies and clinical trials have found that relatively high dietary fiber and whole grain intakes reduce all-cause and cardiovascular-related mortality, the incidence of type 2 diabetes and colorectal cancer, body weight, systolic blood pressure and total cholesterol, and there is dose-response evidence. The relationship with several non-communicable diseases may be causal.	*The Lancet*	533	2019
5th	Greater whole grain intake is associated with lower risk of type 2 diabetes, cardiovascular disease, and weight gain	This meta-analysis shows that whole grain and high fiber intake can prevent vascular disease and reduce the risk of type 2 diabetes and weight gain.	*Journal of Nutrition*	485	2012
6th	Gut microbiome composition is linked to whole grain-induced immunological improvements	This study shows that short-term intake of whole grains can cause changes in intestinal microflora. Whole wheat barley and brown rice increase the abundance of probiotics and improve immune response.	*ISME Journal*	343	2013
7th	Whole grain and refined grain consumption and the risk of type 2 diabetes: a systematic review and dose-response meta-analysis of cohort studies	This systematic review and dose-response meta-analysis showed that whole wheat bread, whole wheat cereals, wheat bran, and brown rice were negatively associated with type 2 diabetes, and white rice increased the risk of diabetes.	*European Journal of Epidemiology*	329	2013
8th	Nutrients, foods, and colorectal cancer prevention	This review focuses on diets to prevent colorectal cancer caused by immune reactivity and risk factors for inflammation, overnutrition, and obesity. Calcium, fiber, milk, and whole grains reduce colorectal cancer risk.	*Gastroenterology*	315	2015
9th	A prospective study of long-term intake of dietary fiber and risk of Crohn’s disease and ulcerative colitis	Long-term intake of dietary fiber, particularly fruit fiber, is associated with a lower risk of Crohn’s disease, but not ulcerative colitis. Fiber from grains, whole grains or legumes does not change disease risk.	*Gastroenterology*	304	2013
10th	Adherence to Mediterranean diet and risk of cancer: an updated systematic review and meta-analysis	In this systematic review and meta-analysis, reduced risk of cancer death appears to be most attributable to fruits, vegetables and whole grains, especially colorectal cancer.	*Nutrients*	299	2017

## Data Availability

Data are contained within the article or Supplementary Material.

## Supplementary Materials

The following supporting information can be downloaded at: https://www.mdpi.com/article/10.3390/foods11244094/s1, Figure S1: Flowsheet of the bibliometric analysis; Figure S2: Time view of keywords (A) and burst map (B) of keywords in whole grain health studies; Table S1: Search strategy; Table S2: Cooperation and contributions of countries/regions to publications; Table S3: Cooperation and contributions of institutions to publications.
