# Supplementary material for: Health Effects of Whole Grains: A Bibliometric Analysis
Source: Foods. 2022 Dec 18;11(24):4094. doi: 10.3390/foods11244094 (PMC9777732; doi:10.3390/foods11244094)
Supplement: Supplementary file 1 [file foods-11-04094-s001.zip › Supplementary Figure.pdf]

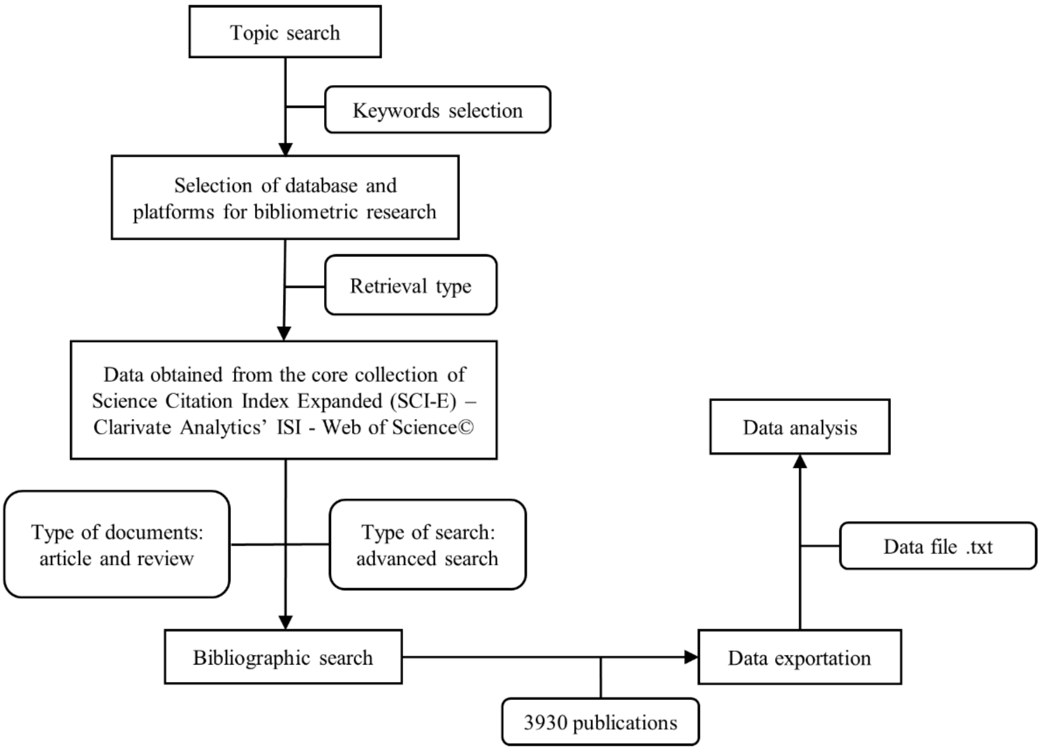

Figure S1. Flowsheet of the bibliometric analysis.

A

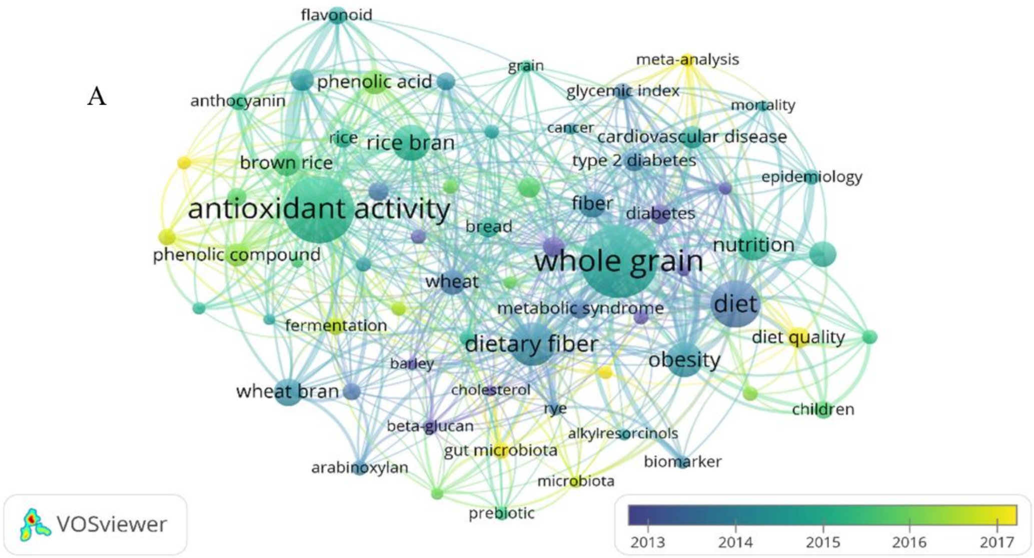

B

Top 25 Keywords with the Strongest Citation Bursts

Keywords	Year	Strength	Begin	End
coronary heart disease	2000	34.75	**2000**	2010
heart disease	2000	11.87	**2000**	2008
glycemic load	2000	10.38	**2000**	2008
fat	2000	7.45	**2000**	2005
cancer	2000	7.41	**2000**	2003
dietary fiber	2000	7.25	**2000**	2009
rat	2000	6.95	**2000**	2011
iowa womens health	2000	6.85	**2000**	2008
absorption	2000	6.62	**2000**	2011
whole grain intake	2000	11.29	**2001**	2007
whole grain consumption	2000	7.89	**2001**	2012
oat bran	2000	8.79	**2002**	2009
risk factor	2000	7.17	**2002**	2011
diabetes mellitus	2000	10.68	**2003**	2011
carbohydrate	2000	6.88	**2003**	2009
vegetable	2000	8.27	**2004**	2010
vitamin e	2000	9.07	**2005**	2015
barley	2000	7.15	**2006**	2011
c reactive protein	2000	6.72	**2006**	2013
weight gain	2000	9.63	**2007**	2013
validity	2000	7.69	**2011**	2015
nutrient intake	2000	6.91	**2011**	2016
cardiovascular risk	2000	6.94	**2012**	2015
white rice	2000	10.32	**2014**	2018
bioactive compound	2000	9.74	**2018**	2021

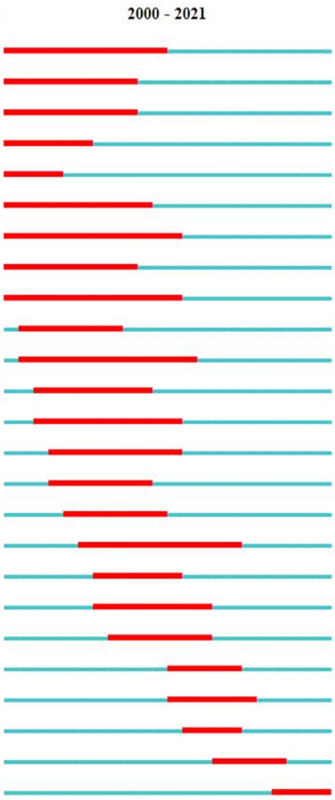

Figure S2. Time view of **(A)** keywords and **(B)** burst map of keywords in whole grain health studies.
