# Supplementary material for: Health Effects of Whole Grains: A Bibliometric Analysis
Source: Foods. 2022 Dec 18;11(24):4094. doi: 10.3390/foods11244094 (PMC9777732; doi:10.3390/foods11244094)
Supplement: Supplementary file 1 [file foods-11-04094-s001.zip › Supplementary Table.pdf]

**Table S1.** Search strategy.

| |
|---|
| #1: "grain*, whole" OR "whole-grain*" OR "grain cereal*, whole" OR "whole-amaranth" OR "whole-barley" OR "whole-buckwheat" OR "whole-corn" OR "whole-millet" OR "whole-oats" OR "whole-quinoa" OR "brown rice" OR "whole-sorghum" OR "whole-teff" OR"whole-triticale" OR "whole-wheat" OR "whole-rice" OR "whole-wild rice" |
| #2: bran OR "rice germ" OR "corn germ" OR "wheat germ" OR endosperm |
| #3: diabete OR "blood glucose" OR "blood sugar" OR insulin OR cardiovascular diseases OR metabolize OR metabolic syndrome OR hypertension OR "blood pressure" "blood lipids" OR "serum lipid" OR coronary OR heart disease OR cholesterol OR obesity OR "body weight" OR fat OR gout OR cancer OR tumor OR intestinal OR gut OR microbiome OR "enteric organisms" OR constipation OR diarrhea OR anti-inflammatory OR inflammation OR antioxidant OR oxidative OR brain OR health |
| #4: feed OR fungus OR "heavy metal" OR contamination OR pollution OR grow OR plant OR arsenic OR cadmium OR rheolog* OR oil OR pasting OR physic* OR "pesticide residu*" OR carcass OR egg* |
| #5: #1 OR #2 AND #3 NOT #4 |

**Table S2.** Cooperation and contributions of countries/regions to publications.

| Rank | Count | Centrality | Year | Country/Region |
|---|---|---|---|---|
| 1 | 1188 | 0 | 2000 | USA |
| 2 | 626 | 0.06 | 2005 | PEOPLES R CHINA |
| 3 | 212 | 0.03 | 2002 | ITALY |
| 4 | 207 | 0 | 2001 | SOUTH KOREA |
| 5 | 206 | 0.02 | 2002 | ENGLAND |
| 6 | 203 | 0 | 2000 | CANADA |
| 7 | 201 | 0 | 2002 | SWEDEN |
| 8 | 169 | 0.12 | 2000 | JAPAN |
| 9 | 152 | 0 | 2007 | INDIA |
| 10 | 148 | 0.12 | 2000 | AUSTRALIA |
| 11 | 146 | 0.06 | 2000 | DENMARK |
| 12 | 127 | 0.44 | 2001 | SPAIN |
| 13 | 127 | 0.24 | 2001 | FINLAND |
| 14 | 117 | 0 | 2003 | GERMANY |
| 15 | 105 | 0 | 2009 | BRAZIL |
| 16 | 102 | 0.18 | 2008 | THAILAND |
| 17 | 96 | 0 | 2005 | IRAN |
| 18 | 95 | 0 | 2001 | NETHERLANDS |
| 19 | 83 | 0.3 | 2001 | FRANCE |
| 20 | 58 | 0.11 | 2001 | NORWAY |
| 21 | 56 | 0.06 | 2010 | TURKEY |
| 22 | 55 | 0.06 | 2006 | MEXICO |
| 23 | 54 | 0.49 | 2003 | SWITZERLAND |
| 24 | 49 | 0.1 | 2010 | MALAYSIA |
| 25 | 47 | 0.12 | 2009 | POLAND |
| 26 | 44 | 0 | 2008 | BELGIUM |
| 27 | 42 | 0.07 | 2007 | PAKISTAN |
| 28 | 28 | 0 | 2010 | GREECE |
| 29 | 28 | 0.18 | 2008 | EGYPT |
| 30 | 25 | 0.11 | 2004 | NEW ZEALAND |

**Table S3.** Cooperation and contributions of institutions to publications.

| Rank | Count | Centrality | Year | Institution |
|---|---|---|---|---|
| 1 | 123 | 0.18 | 2000 | Harvard Univ |
| 2 | 101 | 0.21 | 2000 | Univ Minnesota |
| 3 | 84 | 0.27 | 2002 | Tufts Univ |
| 4 | 72 | 0.09 | 2005 | Univ Copenhagen |
| 5 | 70 | 0.27 | 2000 | Brigham & Womens Hosp |
| 6 | 48 | 0.02 | 2017 | Harvard TH Chan Sch Publ Hlth |
| 7 | 44 | 0.04 | 2006 | Lund Univ |
| 8 | 43 | 0.15 | 2003 | Swedish Univ Agr Sci |
| 9 | 38 | 0.27 | 2002 | Univ Helsinki |
| 10 | 33 | 0.12 | 2011 | Univ Eastern Finland |
| 11 | 26 | 0 | 2009 | Jiangnan Univ |
| 12 | 21 | 0.04 | 2017 | Harvard Med Sch |
| 13 | 21 | 0 | 2015 | Chalmers Univ Technol |
| 14 | 21 | 0 | 2009 | Beijing Technol & Business Univ |
| 15 | 19 | 0 | 2016 | Univ Wollongong |
| 16 | 16 | 0.1 | 2005 | Karolinska Inst |
| 17 | 16 | 0 | 2013 | Isfahan Univ Med Sci |
| 18 | 16 | 0 | 2000 | Gen Mills Inc |
| 19 | 16 | 0.04 | 2011 | Aarhus Univ |
| 20 | 15 | 0.02 | 2016 | Univ Tehran Med Sci |
| 21 | 13 | 0 | 2000 | USDA ARS |
| 22 | 13 | 0 | 2009 | ARS |
| 23 | 13 | 0.02 | 2012 | VTT Tech Res Ctr Finland |
| 24 | 12 | 0.04 | 2000 | Univ Toronto |
| 25 | 12 | 0 | 2016 | Zhejiang Univ |
| 26 | 10 | 0.02 | 2017 | Univ Washington |
| 27 | 10 | 0 | 2007 | Univ Manitoba |
| 28 | 10 | 0 | 2013 | Univ Putra Malaysia |
| 29 | 10 | 0.05 | 2001 | Univ Oslo |
| 30 | 10 | 0 | 2010 | Cornell Univ |
